# Quantitative proteomic analysis of the microbial degradation of 3-aminobenzoic acid by *Comamonas* sp. QT12

**DOI:** 10.1038/s41598-022-17570-9

**Published:** 2022-10-20

**Authors:** Shuxue Zhao, Chao Pan, Junxing Zhao, Haiyan Du, Min Li, Hao Yu, Xi Chen

**Affiliations:** 1 College of Marine Life Sciences, Ocean University of China, Qingdao, 266100 China; 2 Shandong Provincial Key Laboratory of Applied Mycology, School of Life Sciences, Qingdao Agricultural University, 700 Changcheng Road, Chengyang District, Qingdao, 266109 Shandong Province People’s Republic of China; 3 Qingdao Water Administration Bureau, Qingdao, 266071 China

**Keywords:** Biochemistry, Biological techniques, Biophysics, Cell biology, Genetics, Molecular biology

## Abstract

A *mab* cluster associated with 3-aminobenzoic acid (3AB) degradation was identified in *Comamonas* sp. QT12. However, the cellular response of *Comamonas* sp. QT12 to 3-aminobenzoic acid remains unclear. In this study, label-free quantitative proteome analysis based on LC–MS/MS was used to study the protein expression difference of strain QT12 under the condition of using 3AB (3AB) and citric acid/ammonium chloride as substrates (3ABCon). A total of 2068 proteins were identified, of which 239 were significantly up-regulated in 3AB group, 124 were significantly down-regulated in 3AB group, 624 were expressed only in 3AB group, and 216 were expressed only in 3ABCon group in 3AB group. KEGG pathway analysis found that 83 pathways were up-regulated and 49 pathways were down-regulated, In GO analysis, 315 paths were up-regulated and 156 paths were down-regulated. There were 6 genes in the *mab* cluster that were only detected in the 3AB group.The *mab* cluster was found to be related to degradation of 3AB. By knockout, it was found that the growth rate of the mutant △*orf*7 and △*orf*9 were slowed down. HPLC results showed that the mutant △*orf*7 and △*orf*9 could still degrade 3AB, it was found that *orf7*, *orf9* were not key genes about 3AB degradation and they could be replaced by other genes in strain QT12. These findings improve our understanding of the molecular mechanisms underlying the cellular response of 3AB degradation in *Comamonas* bacterium.

## Introduction

Aniline and its derivatives are widely used in the organic synthesis of synthetic dyes, plasticizers, pesticides, herbicides, and drugs^1^. Along with the production, sales, and degradation of these compounds, a large number of aniline substances are discharged into the natural environment. These compounds are carcinogenic, teratogenic, and mutagenic, causing serious harm to the natural environment, humans, and other organisms^2,3^. Benzoic acid is not only a raw material involved in the biosynthesis of the above chemicals but also an important intermediate metabolite of other aniline substances. For example, the degradation of synthetic dyes mordant yellow and mordant orange produces 5-amino salicylic acid (5ASA)^2–5^. Microbial degradation is one of the most effective means to eliminate such pollutants in the environment, which has the advantages of high efficiency, mild conditions and no secondary pollution. At present, many 3-aminobenzoic acid (3AB) degrading strains have been isolated and studied, including *Pusillimonas*, *Methanothrix*, *Comamonas* et al^6–8^. Proteomics has been widely used in the qualitative and quantitative analysis of proteins in biological samples, and LC–MS/MS is a useful mean for quantitative proteomics studies^9,10^. Proteomic analysis can further understand and analyze the complex metabolic pathways and related proteins in cells^9–11^, the approach has been used to parse metabolic pathways and screen metabolic genes associated with metabolic pathways^12–14^. Changes in microbial adaptive responses to different substrates or environments can also be analyzed by high-throughput proteomics^15–19^.

In the previous study, we isolated a strain of *Comamonas* sp QT12, the *mab* cluster related to 3AB degradation was found, and the function of some genes on the *mab* cluster were analyzed. To further study the correlation between other genes on the *mab* cluster and 3AB degradation, strain QT12 was cultured in liquid media in the presence or absence of 3AB were comparatively analyzed. The significantly differentially expressed proteins in the proteomic profile were searched to clarity the molecular mechanism involved in 3AB degradation in strain QT12.

## Materials and methods

### Chemicals

3-aminobenzoic acid, citric acid, ammonium chloride, PBS buffer purchased from Shenggong Bioengineering (Shanghai, China). Reagents such as formic acid, trifluoroacetic acid, ammonium bicarbonate, urea, Tris-HCl, and methanol were purchased purely from ThermalFisher for LC–MS. Sequencing grade trypsin was purchased from Promega. HaltTM Protease Inhibitor Cocktail, Pierce Protein Concentrators PES (10K MWCO, 0.5 mL) purchased from ThermalFisher. Merck Millipore ZipTip C18 Resin, a desalinized column, was purchased from Darmstadt.

### Medium and culture conditions

MSM medium reference^20^, the final concentration of 3AB was 1000 mg/L, pH was adjusted to 7.0, and labeled as 3AB group. The final concentration of citric acid/ammonium chloride was 1000 mg/L, pH was set to 7.0, labeled as 3ABCon group, sterilized at 115 °C for 30 min.

Strain *Comamonas* sp. QT12 was cultured in a triangular flask with 3AB group and 3ABCon group at 30 °C, 125 rpm, and darkness. *Escherichia coli* was cultured in LB broth at 37 °C, 125 rpm, and darkness.

### Proteome sample preparation and proteolytic hydrolysis

Strain QT12 was cultured on 3AB group medium and 3ABCon group medium, respectively, and the same medium was used for the continuous passage more than three times. Strains cultured at 10 mL to the middle logarithmic stage (OD_600_ between 1.5 and 2.0) were centrifuged at 8000×*g* for 5 min to collect the strains. The strains were washed twice with PBS, and the PBS buffer was removed by centrifugation. 1 mL lysis buffer (2% SDS, 0.1 mol/L Tris-HCI, pH 7.6, 1 × protease inhibitor complex) was added to the cells, and the QT12 were suspended, transferred to a 2 mL centrifuge tube, and two 5 mm steel strains were added. After precooling, the cells were grinded with TissueLyser II tissue grinder (QIAGEN, Hilden, Germany) at 300 Hz for 1 min, and repeated once. After grinding, the protein samples were centrifuged at 10,000×*g* for 2 min, and the supernatant was transferred to a new centrifuge tube, ultrasonic for 24 s (ultrasonic for 6 s in 4 times, the interval for 15 s). After centrifugation at 12,000 rpm for 10 min, the supernatant was transferred to a new 1.5 mL centrifuge tube, and the protein concentration was measured using a BCA protein determination kit. Each protein sample was added with 15 mg DTT and incubated at 56 °C for 1 h to reduce the protein.

100 μg protein sample was added to a 300 μL UA buffer (8 mol/L urea dissolved in 0.1 mol/L Tris-HCI, pH 8.5), and then diluted into the FASP ultrafiltration tube, centrifuged at 10,000×*g* for 30 min. The protein was washed once with 300 μL UA Buffer. 100 μL UA Buffer containing 50 mM IAA was added to the centrifuge tube, mixed with an oscillator for 1 min, and incubated for 30 min in darkness. The protein was centrifuged at 10,000×*g* for 15 min and washed 3 times with 200 μL UA buffer. Add 300 μL of 50 mM ammonium bicarbonate buffer to the ultrafiltration tube, centrifuge at 10,000×*g* for 10–15 min, repeat twice. 2 μg trypsinase was added to 100 μL of 50 mM ammonium bicarb buffer solution, and then added to the ultrafiltration tube. The ultrafiltration tube was oscillated for 1 min by the vortex. The ultrafiltration tube was placed in the oscillating gas bath shaker at 37 °C, and the enzyme was hydrolyzed overnight. The ultrafiltration tube was transferred to a new collection tube and centrifuged at 10,000×*g* for 15 min. The enzymatic hydrolysis peptides were collected and buffered with 50 μL 50 mM ammonium bicarbonate for 2 times, and the filtrate was combined. The reaction was terminated by adding trifluoroacetic acid to the final concentration of 0.4%. The peptide concentration was determined using the peptide determination kit.

The C_18_ column was activated by adding 200 μL methanol, centrifugation at room temperature at 1200*g* for 10 min, washing twice with 190 μL 80% acetonitrile /0.2% trifluoroacetic acid buffer, and then washing three times with 0.2% trifluoroacetic acid. 20 μg peptide was diluted to 190 μL with 0.2% trifluoroacetic acid. The diluted peptide sample was added to the desalting column and centrifuged at 2000*g* for 12 min. After washing with 0.2% trifluoroacetic acid three times, the desalting column was transferred to a new centrifugal tube. Add 180 μL 80% acetonitrile/0.2% trifluoroacetic acid buffer, wash twice, and combine the effluent. Dry in centrifugal concentrator at room temperature and store to − 80 °C.

### LC–MS /MS detection and data analysis

The LC–MS /MS analysis method was completed by the Central Laboratory of Qingdao Agricultural University. Lyophilized peptide was dissolved with 10 μL 0.1% (V/V) formic acid. The nano-LC system was combined with Orbitrap Fusion Tribrid (ThermoFisher, CA, USA) for tag-free quantitative proteomic analysis. As mentioned earlier^21^, data collection is carried out in a data-dependent collection mode. Each group had 2 biological replicates.

The original protein expression was standardized by the method of summation. This Whole Genome Shotgun project of strain QT12 has been deposited at DDBJ/ENA/GenBank under the accession MZNW00000000. The version described in this paper is version MZNW01000000.The independent sample T-test was carried out by using a bilateral test. Enrichment analysis of differential proteins was performed using the GO database and KEGG metabolic pathway database^22^. Protein interaction analysis was performed using STRING database^23^. Data analysis and mapping using online analytical tools wukong cloud platform (https://www.omicsolution.com/wkomics/main/) and ggplot2 was complete.

### *Orf7* and *orf9* gene knockout and functional verification

In order to verify the role of *mab* cluster genes of the QT12 strain in 3AB metabolism, the functions of *mabA* and *mabB* genes have been verified^6,20^. The function were predicted according to the sequence of *orf7* and *orf7*, and its function were verified by gene knockout^6^. The degradation of 3AB by mutant strains △*orf*7, △*orf*9 and wild-type strain QT12 were compared to verify whether their played a key role in the degradation of 3AB. In this study, primer sequences were shown in Table 1.

**Table 1:** PCR primers used in this study.

Name	Sequence (5′–3′)
*Orf7*-AW-F	ATGACATGATTACGAATCTCGGTGTATACCCGCGT
*Orf7*-AW-R	TCCGGGACGATGCCAACGTGCTCGGGCCGTCAGGC
*Orf7*-BW-F	GCCTGACGGCCCGAGCACGTTGGCATCGTCCCGGA
*Orf7*-BW-R	CCGGGTACCGAGCTCGAATGCGTGCAATTCTGAGCA
*Orf9*-AF-E	CCGGAATTCTTGAACATGCTCAGAATTGCA
*Orf9*-AR-X-2	CCGCTCGAGACGCGAGGCCTTAGCAGTGGC
*Orf9*-BF-X	CCGCTCGAGTCCCTTCGGGCCCTGGCTGG
*Orf9*-BR-B	CGCGGATCCTCACTCTTGTGCGACCGTATT

The strains of △*orf*7, △*orf*9, and wild type QT12 growth curves in 3AB medium were measured in order to find the differences of in growth conditions. Resting cell reaction of the three strains were used to detect the intermediates and products of 3AB degradation. 3AB was used as the only carbon and nitrogen source to culture the three strains. After 24 h, the bacteria were collected by centrifugation at 8000 rpm for 5 min, washed by Tris-HCl (50 mmol, pH7.0) for three times, and the OD_600_ absorbance value was determined. The final absorbance value (OD_600_) of three strains were set to 10. The concentration of 3AB was 0.5 mg/mL, the strains cultured at 30 °C with three parallel cells in each group. In this study, took samples every 4 h, samples were centrifuged at 12,000 rpm for 5 min, then took 200 μL supernatant into a new 1.5 mL centrifuge tube. Added 4 times the volume of methanol to supernatant, samples centrifuged at 12,000 rpm for 5 min, filtered samples into liquid vial with 0.22 μm filter membrane for HPLC detection. The conditions of HPLC were 95% 0.6 mM ammonia solution 5% methanol at a flow rate of 0.5 mL/min, column temperature 30 °C.

## Results

### Proteome analysis

**Figure 1:**
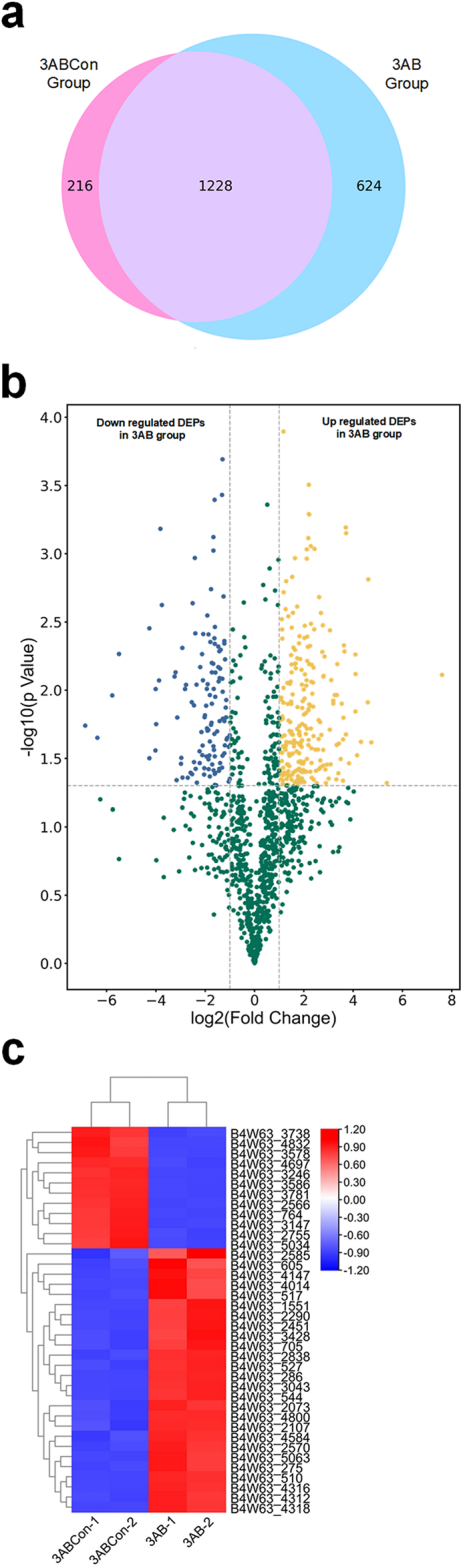
Analysis of the difference between protein quantity and protein produced by cultured QT12 in 3AB group and 3ABCon group. (**a**) Venn diagram analysis of the proteins in 3AB group and 3ABCon group. (**b**) Volcano plot analysis of the identified proteins showing the DEPs. Blue plots, downregulated DEPs in 3AB group; Yellow plots, upregulated DEPs in 3AB group; Green plots, proteins without differential expression. (**c**) Results of heat map analysis of differential protein of strain QT12 under 3AB group and 3ABCon group.

A total of 2068 proteins were detected by LC–MS/MS analysis in 3AB group and 3ABCon Group. Among them, 624 proteins were expressed only in 3AB group and 216 proteins were expressed only in 3ABCon Group (Fig. 1a, Table S1). It can be seen from the results that the number of proteins detected only in the presence of 3AB was significantly higher than that in the control group, indicating that the protein expression of the QT12 strain changed dramatically to adapt to the degradation process of 3AB. A 2.0-fold change cut-off and *p* value < 0.05 were used to categorize proteins with differential abundances (DAPs). Compared with 3ABCon group, 3AB up-regulated 239 and down-regulated 124 the protein (Fig. 1b). Fold change > 10 or < 0.1 differential proteins were selected. Through heat map analysis, it was found that protein expression in 3AB group and 3ABCon group was significantly different (Fig. 1c, Table S2), which can be found that there were more differential proteins in strain QT12 under two different culture conditions.

**Figure 2:**
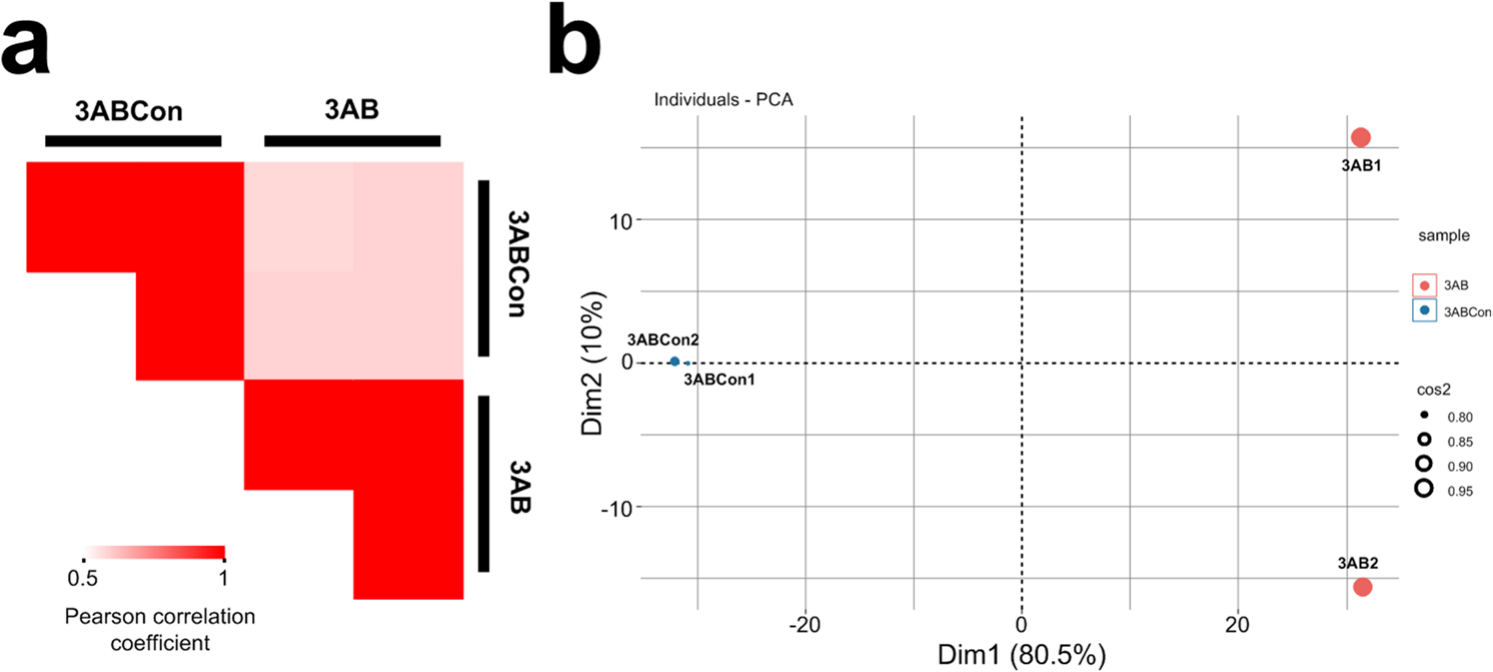
Correlation analysis and PCA of identified proteins in 3AB group and 3ABCon group. (**a**) The matrix of correlation plots revealed correlations between samples from 3AB group and 3ABCon group. (**b**) PCA of proteins detected in 3AB group and 3ABCon group.

In order to test the parallelism of proteome samples and the differences among different samples, correlation analysis, and PCA analysis were performed. The results show that the correlation coefficient between two samples from the same group was very high between 0.99 and 1.0, while the similarity coefficient between samples from different groups was very low between 0.58 and 0.59 (Fig. 2a). PCA analysis also showed similar results. The first two principal components accounted for 90.5% of the weight, and the samples of 3AB and 3ABCon could be clearly distinguished by the first principal component (Fig. 2b, Table S3). This indicates that the parallelism among samples of the same group was good, and there were significant differences among different samples. Proteome data could reflect the protein expression difference of strain QT12 under different substrate metabolism.

### Cluster analysis of differential proteins

Through the enrichment of the function of the differential proteins, the changes of the protein profile of strain QT12 caused by the changes of the culture medium can be better displayed. According to the GO classification, the proteins with significant differences in expression levels between the two groups were clustered, and the proteins were divided into three categories: gene or protein molecular function (MF), constituent cell component (CC), and biological process (BP).

**Figure 3:**
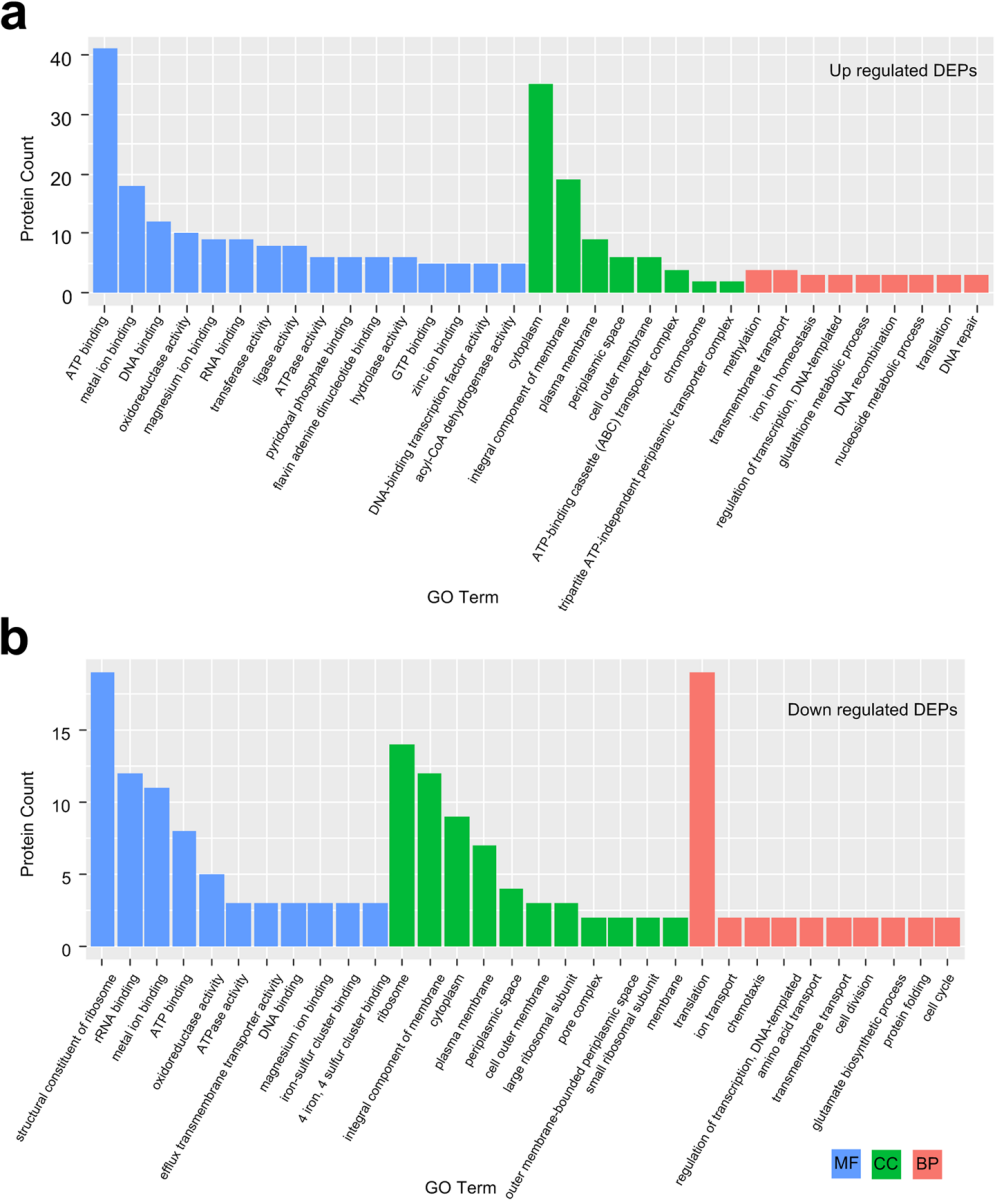
Bar graph of Gene Ontology (GO) enrichment analysis. (**a**) Most enriched GO terms of up-regulated DEPs in 3ABgroup versus 3AB Con group. (**b**) Most enriched GO terms of down-regulated DEPs in 3AB group and 3AB Con group.

According to GO clustering results, 3AB-up DAPs mainly found ATP binding, metal ion binding, and DNA binding in the category of molecular functions, including 356 up-regulated pathways. Among cell components, 3AB-up DAPs are mainly enriched in the cytoplasm, an Integral component of membrane and plasma membrane, including 89 up-regulated pathways. In biological processes, 3AB-up DAPs are mainly associated with methylation, transmembrane transport, and iron ion homeostasis, including 172 up-regulated pathways (Fig. 3a). The downdraft of DEPs mainly exists in structural Constituent of Ribosome, Ribosome, translation, and other processes (Fig. 3b). 3AB-down DAPs mainly found structural constituent of the ribosome, rRNA binding, metal ion binding in the category of molecular functions, including 166 down-regulated pathways. Among cell components, 3AB-down DAPs are mainly enriched in ribosome, integral component of membrane and cytoplasm, including 66 down-regulated pathways. In biological processes, 3AB-down DAPs are mainly associated with translation, ion transport, and chemotaxis,including 73 down-regulated pathways (Fig. 3b, Table S4).

**Figure 4:**
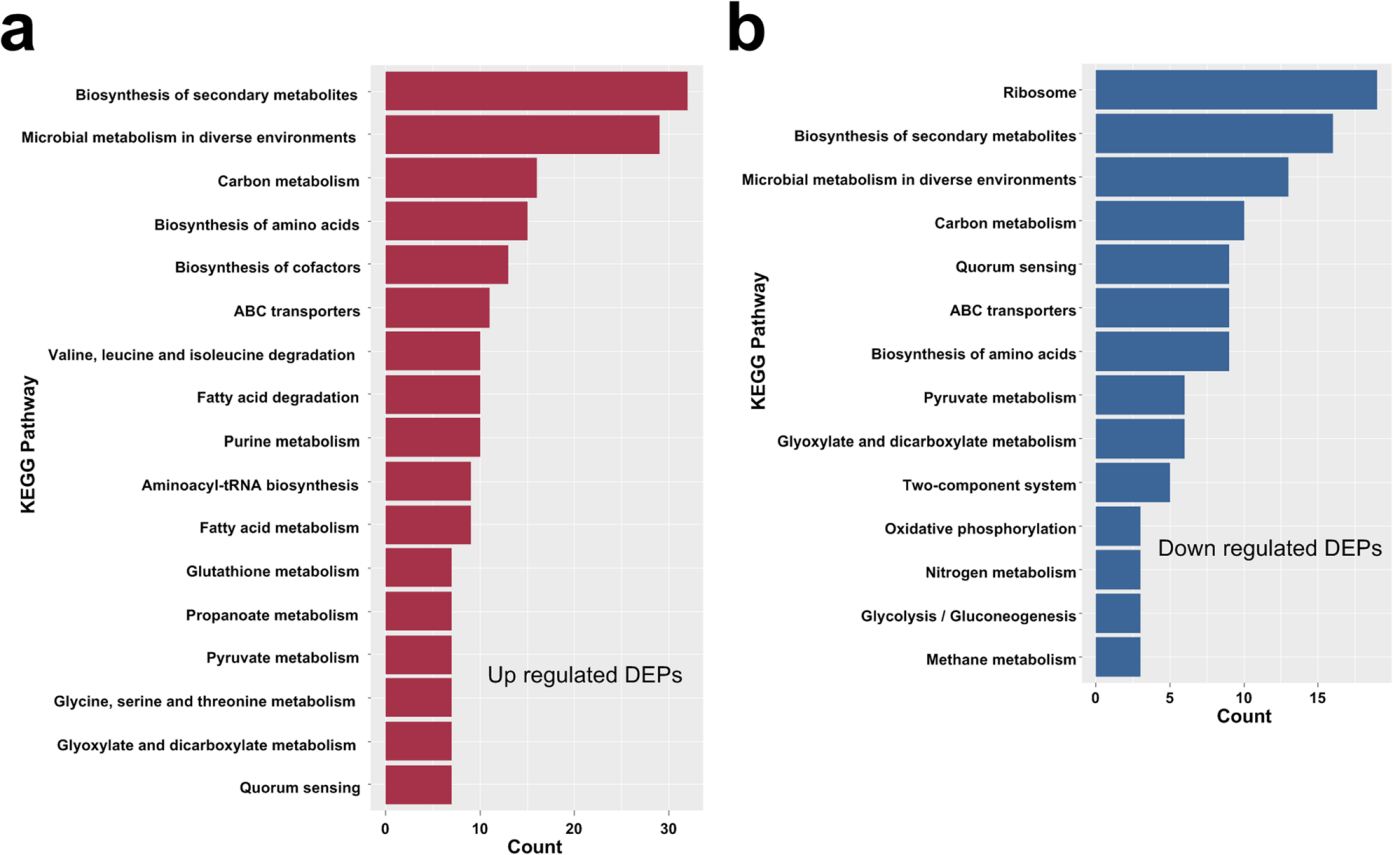
Bar graph of KEGG enrichment analysis. (**a**) Most enriched KEGG pathways of up-regulated DEPs in 3AB group and 3ABCon group. (**b**) Most enriched KEGG pathways of down-regulated DEPs in 3ABgroup and 3ABCon group.

KEGG analysis found that Biosynthesis of secondary metabolites、Microbial metabolism in diverse environments、Carbon metabolism、Biosynthesis of amino acids、Biosynthesis of cofactors、ABC transporters et al. pathways were up-regulated and Ribosome、Biosynthesis of secondary metabolites、Microbial metabolism in diverse environments、Carbon metabolism、Quorum sensing、Biosynthesis of amino acids et al. pathways were down-regulated. KEGG enrichment analysis showed that up-regulated DEPs and down-regulated DEPs shared several pathways such as biosynthesis of secondary metabolism, microbial metabolism in diverse environments, carbon metabolism, The pathways of Aminoacyl-Trna biosynthesis, Glutathione Metabolism, Glycine serine and threonine metabolism, etc. were only enriched by up-regulated DEPs (Fig. 4a). While, Ribosome, Oxidative phosphorylation, and Bacterial chemotaxis etc. were only enriched by down-regulated DEPs (Fig. 4b, Table S5).

### Functions analysis of *mab c*luster genes

**Figure 5:**
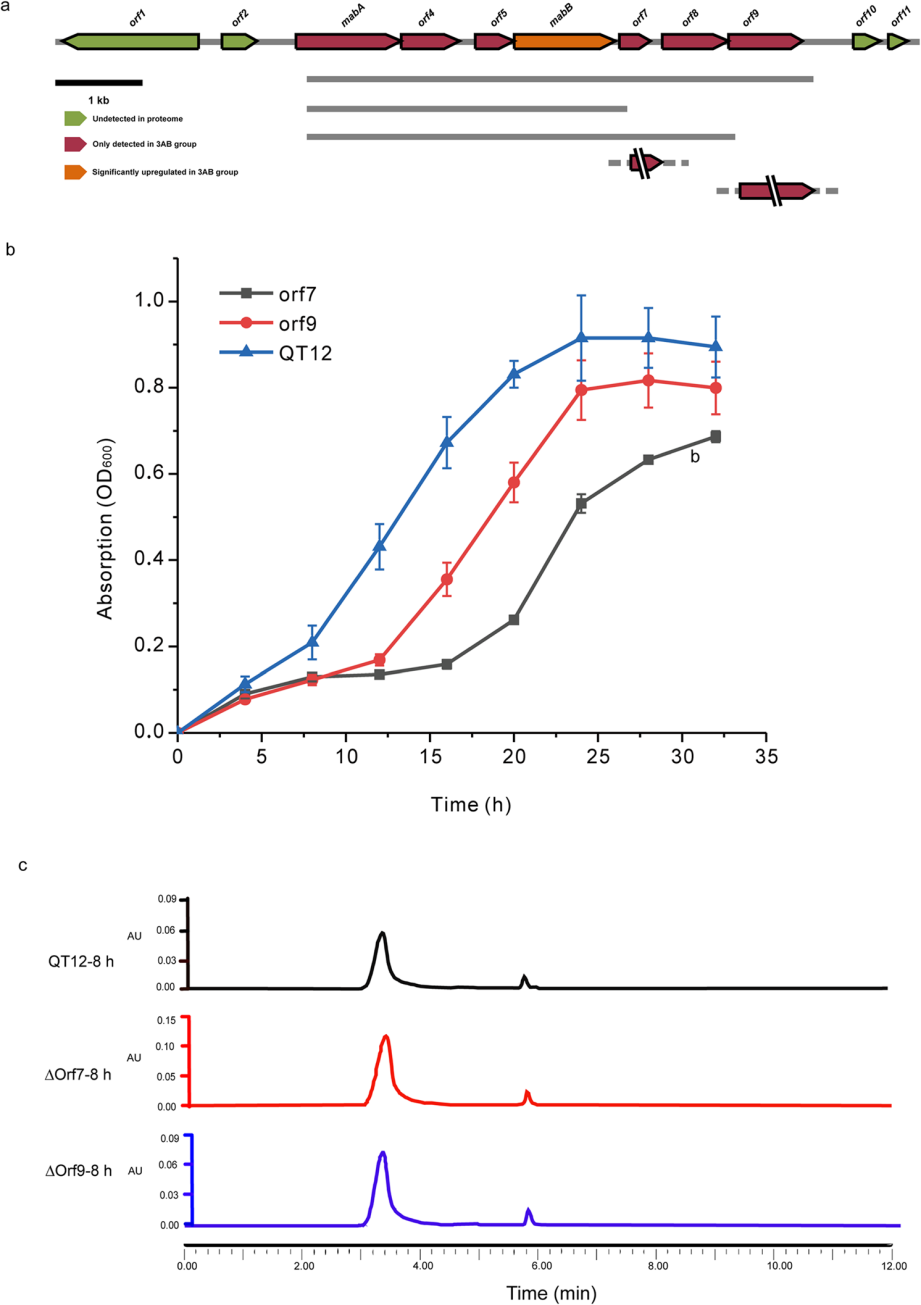
Effect of *mab* cluster on degradation of 3AB and determination of growth metabolic pathway of △*orf7* and △*orf9* mutant strains. (**a**) Physical map of the DNA fragment containing *mab* cluster involved in 3AB degradation in *Comamonas* sp. QT12. The arrows indicates the location, direction and size of the transcription of the orfs. (**b**) The growth of wild-type strain QT12, △*orf7* and △*orf9* mutant strains on 3AB group. (**c**) The metabolites of the wild-type strain QT12, △*orf7* and △*orf*9 mutant strain products were detected by HPLC.

Analysis of proteomic data revealed that 6 of the 11 genes in the *mab* cluster encode proteins that were detected in 3AB group, including *mabA*, *orf4, orf5, orf7, orf8 , orf9* (Fig. 5a).

Among the 7 proteins encoded by *mabA* to *orf9* genes, the expression levels MabB proteins in group 3AB was significantly higher than that in group 3ABCon (194.50 times). The remaining 6 proteins were detected only in group 3AB, which verified our previous speculation that *mab* cluster was closely related to the degradation of 3AB, and we further confirmed that the genes involved in the degradation of 3AB were the 7 genes from *mabA* to *orf9*.

The regulatory protein encoded by *orf8* may be involved in the transcriptional regulation process of the entire *mab* cluster, while the deaminase encoded by *orf7* and hydrolase encoded by *orf9* may be speculated to be involved in the subsequent degradation of cis-ACOHDA, and cis-ACOHDA may produce fumarpyruvate under the action of deaminase. It is further catalyzed by hydrolases to form fumaric acid and pyruvate. To verify this prediction, we used the homologous recombination method to delete *orf*7 and *orf9* genes without a trace and obtained mutant △orf*7* and △*orf9*. The growth rate of the two mutant strains and wild-type QT12 were measured at 3AB group, and it was found that wild-type QT12 had the shortest lag time and the best growth, followed by △*orf9*. When *orf7* was knocked out, it was found that the growth of the strain was affected, and the time to enter the logarithmic growth phase was prolonged, and the growth of the strain was affected (Fig. 5b).

The three strains degradated of 3AB products measured by HPLC. The results of HPLC showed that the wild-type strain QT12 and △*orf7* and △*orf9* strain could produce cis-ACOHDA (RT 3.4 min) and 5-aminosalicylic acid (RT 5.8 min) during the degradation of 3AB (supplementary materials Fig. 5c) at 354 nm. These results indicated that the three strains had the same upstream pathway of 3AB degradation. These results indicated that *orf7* and *orf9* were indeed involved in the degradation of 3AB, but there were other deaminases and hydrolases in strain QT12 that could also fulfill the functions of the proteins encoded by these two genes.

## Discussion

The simple monocyclic amino-aromatic acids degradation is essential to detoxify these pollutants in wastewaters and contaminated soils^1^. While the degradation of 2-aminobenzoate and 4-aminobenzoate has been extensively reported, and the process for the degradation of these two amino-aromatic acids have been characterized^24–28^. The molecular mechanism and pathway of 3AB degradation have also been reported^6,8^. however the molecular mechanism is not fully understood.

In this work, we used unlabeled proteomic analysis to investigate the mechanism of *Comamonas* sp.QT12 cellular response to 3AB. Efficient degradation of 3AB requires the expression not only of enzymes directly involved in the catalytic reaction of 3AB conversion but also of corresponding enzymes, such as cofactors and energy-producing enzymes. A total of 2068 proteins were identified, of which 239 were significantly up-regulated in 3AB group, 124 were significantly down-regulated in 3AB group, 624 were expressed only in 3AB group, and 216 were expressed only in 3ABCon group in 3AB group. In the *mab* cluster, 6 genes were detected only in 3AB group, the expression levels MabB proteins in group 3AB was significantly higher than that in group 3ABCon (194.50 times). The results showed that *mab* cluster was the key gene cluster of 3AB degradation, and the 7 genes from *mabA* to *orf9* were closely related to the degradation of 3 aminobenzoic acid. After *orf7* and *orf9* were knocked out, respectively. The mutant could still grow using 3AB, indicating that *orf7* and *orf9* were not the key genes for 3AB degradation, and there were other genes that could replace *orf7* and *orf9* in strain QT12. The information provided in this study can help us understand and improve the degradation of aniline derivatives by bacteria.

## Supplementary Information

Supplementary Information 1.

Supplementary Information 2.

Supplementary Information 3.

Supplementary Information 4.

Supplementary Information 5.

## Data Availability

The dataset supporting the conclusions of this article is included within the article. All data are fully available without restriction.
